# Supplementary material for: Enzymatic synthesis of mono- and trifluorinated alanine enantiomers expands the scope of fluorine biocatalysis
Source: Commun Chem. 2024 May 9;7:104. doi: 10.1038/s42004-024-01188-1 (PMC11082193; doi:10.1038/s42004-024-01188-1)
Supplement: Supplementary file 2 — Supplementary Information [file 42004_2024_1188_MOESM2_ESM.pdf]

SUPPLEMENTARY INFORMATION

Enzymatic synthesis of mono- and trifluorinated alanine enantiomers expands the scope of fluorine biocatalysis

Manuel Nieto-Domínguez[1], Aboubakar Sako[1], Kasper Enemark-Rasmussen[2], Charlotte Held Gotfredsen[2], Daniela Rago[1], and Pablo I. Nikel*[1]

[1] The Novo Nordisk Foundation Center for Biosustainability, Technical University of Denmark, Kongens Lyngby, Denmark

[2] NMR Center, Department of Chemistry, Technical University of Denmark, Kongens Lyngby, Denmark

e-mail: pabnik@biosustain.dtu.dk

SUPPLEMENTARY METHODS

Table S1. Nucleotide sequences of synthetic gene fragments encoding the enzymes of this work.

Description	Sequence (5'→3')
^{Vp}*ald*<br><br>Encoding the alanine dehydrogenase from *Vibrio proteolyticus*<br><br>(UniProt ID O85596)	ATCATCGGTGTTCCGAAAGAAATCAAAAACCACGAATACCGTGTTGGTATGAT<br>CCCGGCGTCTGTTCGTGAACTGATCTCTCACGGTCACCAGGTTTTCGTTGAAA<br>CCAACGCGGGTGCGGGTATCGGTTTCTCTGACGACGACTACATCGCGGTTGGT<br>GCGTCTATCCTGCCGACCGCGGCGGAAGTTTTCGCGCAGGCGGACATGATCGT<br>TAAAGTTAAAGAACCGCAGGCGGTTGAACGTGCGATGCTGAAAGAAGGTCAGA<br>TCCTGTTCACCTACCTGCACCTGGCGCCGGACTTCCCGCAGACCGAAGACCTG<br>ATCAAATCTAAAGCGGTTTGCATCGCGTACGAAACCGTTACCGACAACATGGG<br>TCGTCTGCCGCTGCTGGCGCCGATGTCTGAAGTTGCGGGTCGTATGTCTATCC<br>AGGCGGGTGCGCAGACCCTGGAAAAATCTCACGGTGGTCGTGGTCTGCTGCTG<br>GGTGGTGTTCCGGGTGTTGAACCGGCGAAAGTTGTTATCGTTGGTGGTGGTGT<br>TGTTGGTGCGAACGCGGCGCGTATGGCGGTTGGTATGCGTGCGGACGTTACCA<br>TCCTGGACCGTAACATCGACACCCTGCGTAAACTGGACGAAGAATTCCAGGGT<br>CGTGCGAAAGTTGTTTACTCTACCGAAGACGCGATCGAAAAACACGTTCTGGC<br>GGCGGACCTGGTTATCGGTGCGGTTCTGATCCCGGGTGCGGCGGCGCCGAAAC<br>TGGTTACCAAAGAACACATCGCGAAAATGAAACCGGGTGCGGCGGTTGTTGAC<br>GTTGCGATCGACCAGGGTGGTTGCTTCGAAACCTCTCACGCGACCACCCACGC<br>GGACCCGACCTACATCGTTGACGACGTTGTTCACTACTGCGTTGCGAACATGC<br>CGGGTGCGGTTGCGCGTACCTCTACCTTCGCGCTGAACAACGCGACCCTGCCG<br>TACATCGTTAAACTGGCGAACAAAGGTTACCGTGAAGCGCTGCTGGCGGACCA<br>CGGTTTCCTGGAAGGTCTGAACGTTATCCACGGTAAAGTTACCTGCAAAGAAG<br>TTGCGGAAGCGTTCAACCTGGAATACGTTCAGCCGGAAACCGCGATCGCGATG<br>TTCAACTAA
St*dapdh*<br><br>Encoding the diaminopimelate dehydrogenase from *Symbiobacterium thermophilum*<br><br>(UniProt ID Q67PI3)	GACAAACTGCGTGTTGCGGTTGTTGGTTACGGTAACGTTGGTCGTTACGCGCT<br>GGAAGCGGTTCAGGCGGCGCCGGACATGGAACTGGTTGGTGTTGTTCGTCGTA<br>AAGTTCTGGCGGCGACCCCGCCGGAACTGACCGGTGTTCGTGTTGTTACCGAC<br>ATCTCTCAGCTGGAAGGTGTTCAGGGTGCGCTGCTGTGCGTTCCGACCCGTTC<br>TGTTCCGGAATACGCGGAAGCGATGCTGCGTCGTGGTATCCACACCGTTGACT<br>CTTACGACATCCACGGTGACCTGGCGGACCTGCGTCGTCGTCTGGACCCGGTT<br>GCGCGTGAACACGGTGCGGCGGCGGTTATCTCTGCGGGTTGGGACCCGGGTAC<br>CGACTCTATCATCCGTGCGCTGCTGGAATTCATGGCGCCGAAAGGTATCACCT<br>ACACCAACTTCGGTCCGGGTATGTCTATGGGTCACTCTGTTGCGGTTAAAGCG<br>ATCCCGGGTGTTCGTGACGCGCTGTCTATGACCATCCCGGCGGGTATGGGTGT<br>TCACAAACGTGCGGTTTACGTTGAACTGGAACCGGGTGCGGACTTCGCGGAAG<br>TTGAACGTGCGATCAAAACCGACCCGTACTTCGTTCGTGACGAAACCCGTGTT<br>ACCCAGGTTGAATCTGTTTCTGCGCTGATGGACGTTGGTCACGGTGTTGTTAT<br>GGAACGTAAAGGTGTTTCTGGTGCGACCCACAACCAGCTGTTCCGTTTCGAAA<br>TGCGTATCAACAACCCGGCGCTGACCGCGCAGGTTATGGTTGCGGCGCTGCGT<br>GCGGCGGCGCGTCAGAAACCGGGTTGCTACACCATGATCGAAATCCCGGTTAT<br>CGACTACCTGCCGGGTGACCGTGAAGCGTGGATCCGTAAACTGGTTTGA
^{Sl}*alr*<br><br>Encoding the alanine racemase from *Streptomyces lavendulae*<br><br>(UniProt ID Q65YW7)	AATGAAACACCTACGCGTGTTTACGCCGAGATCGACTTGGACGCCGTTCGTGC<br>TAACGTACGTGCATTGCGCGCCCGTGCACCCCGCTCGGCGCTGATGGCCGTTG<br>TAAAAAGCAATGCTTACGGCCATGGTGCGGTACCATGCGCTCGTGCAGCGCAA<br>GAAGCAGGGGCAGCATGGTTGGGTACGGCCACGCCTGAGGAGGCGTTAGAACT<br>GCGTGCTGCCGGGATCCAGGGTCGCATTATGTGCTGGCTTTGGACCCCGGGTG<br>GGCCATGGCGTGAGGCGATCGAGACTGACATTGATGTAAGTGTGTCAGGTATG<br>TGGGCATTAGATGAAGTTCGTGCAGCAGCGCGCGCAGCTGGCCGTACTGCGCG<br>TATTCAGTTGAAGGCTGATACAGGACTGGGACGTAATGGTTGTCAGCCTGCGG<br>ACTGGGCTGAACTTGTGGGCGCAGCAGTTGCGGCGCAAGCAGAAGGTACGGTG<br>CAGGTTACTGGAGTATGGTCTCATTTCGCTTGCGCTGATGAACCAGGCCACCC<br>ATCGATTCGCTTACAGCTGGATGCGTTCCGCGATATGTTAGCGTACGCAGAAA

	AGGAAGGCGTAGACCCAGAGGTGCGCCATATCGCTAATAGTCCCGCTACTCTG<br>ACCCTTCCAGAGACGCATTTCGATTTAGTACGTACTGGGCTTGCTGTATACGG<br>AGTAAGTCCTTCTCCTGAATTAGGGACGCCGGCGCAATTAGGCCTTCGCCCCG<br>CTATGACACTGCGTGCGAGTCTGGCGTTAGTCAAAACAGTGCCCGCTGGCCAT<br>GGGGTCTCGTATGGTCACCACTATGTCACCGAGTCGGAGACTCACCTGGCTTT<br>AGTACCAGCGGGATACGCAGATGGGATCCCTCGCAACGCCTCCGGTCGCGGGC<br>CTGTCTTAGTGGCGGGTAAGATTCGCCGCGCTGCTGGCCGCATCGCGATGGAT<br>CAATTCGTTGTAGACCTGGGAGAAGATTTGGCAGAGGCTGGCGATGAGGCCGT<br>TATCCTGGGAGATGCAGAACGCGGTGAACCGACTGCTGAGGACTGGGCTCAAG<br>CCGCCCACACAATTGCATATGAAATTGTAACTCGCATCGGTGGCCGCGTTCCA<br>CGCGTTTACCTGGGTGGTTGA
^{Ec}*alr*<br><br>Encoding the alanine racemase from *Escherichia coli*<br><br>(UniProt ID P0A6B4)	CAGGCGGCGACCGTTGTTATCAACCGTCGTGCGCTGCGTCACAACCTGCAGCG<br>TCTGCGTGAACTGGCGCCGGCGTCTAAAATGGTTGCGGTTGTTAAAGCGAACG<br>CGTACGGTCACGGTCTGCTGGAAACCGCGCGTACCCTGCCGGACGCGGACGCG<br>TTCGGTGTTGCGCGTCTGGAAGAAGCGCTGCGTCTGCGTGCGGGTGGTATCAC<br>CAAACCGGTTCTGCTGCTGGAAGGTTTCTTCGACGCGCGTGACCTGCCGACCA<br>TCTCTGCGCAGCACTTCCACACCGCGGTTCACAACGAAGAACAGCTGGCGGCG<br>CTGGAAGAAGCGTCTCTGGACGAACCGGTTACCGTTTGGATGAAACTGGACAC<br>CGGTATGCACCGTCTGGGTGTTCGTCCGGAACAGGCGGAAGCGTTCTACCACC<br>GTCTGACCCAGTGCAAAAACGTTCGTCAGCCGGTTAACATCGTTTCTCACTTC<br>GCGCGTGCGGACGAACCGAAATGCGGTGCGACCGAAAAACAGCTGGCGATCTT<br>CAACACCTTCTGCGAAGGTAAACCGGGTCAGCGTTCTATCGCGGCGTCTGGTG<br>GTATCCTGCTGTGGCCGCAGTCTCACTTCGACTGGGTTCGTCCGGGTATCATC<br>CTGTACGGTGTTTCTCCGCTGGAAGACCGTTCTACCGGTGCGGACTTCGGTTG<br>CCAGCCGGTTATGTCTCTGACCTCTTCTCTGATCGCGGTTCGTGAACACAAAG<br>CGGGTGAACCGGTTGGTTACGGTGGTACCTGGGTTTCTGAACGTGACACCCGT<br>CTGGGTGTTGTTGCGATGGGTTACGGTGACGGTTACCCGCGTGCGGCGCCGTC<br>TGGTACCCCGGTTCTGGTTAACGGTCGTGAAGTTCCGATCGTTGGTCGTGTTG<br>CGATGGACATGATCTGCGTTGACCTGGGTCCGCAGGCGCAGGACAAAGCGGGT<br>GACCCGGTTATCCTGTGGGGTGAAGGTCTGCCGGTTGAACGTATCGCGGAAAT<br>GACCAAAGTTTCTGCGTACGAACTGATCACCCGTCTGACCTCTCGTGTTGCGA<br>TGAAATACGTTGACTGA

Table S2. Nucleotide sequences of primers used for plasmid construction.

Name	Sequence (5'→3')[a]	Description
pET28a-F	ATCTCTTCuGAGCACCACCACCACC	Amplification of pET-28a(+)-TEV for USER cloning, forward
pET28a-R	ATGGCCCuGAAAATAAAGATTCTCGCCGCT	Amplification of pET-28a(+)-TEV for USER cloning with Nt His-tag, reverse
vpald-F	AGGGCCAuATCATCGGTGTTCCGAAAGA	Amplification of *vpald* for USER cloning into pET28a(+)-TEV in-phase with Nt His-tag, removes ATG, forward
vpald-R	AGAAGAGAuTTAGTTGAACATCGCGATCG	Amplification of *vpald* for USER cloning into pET28a(+)-TEV in-phase with Nt His-tag, reverse
stdapdh-F	AGGGCCAuGACAAACTGCGTGTTGCGGT	Amplification of *stdapdh* for USER cloning into pET28a(+)-TEV in-phase with Nt His-tag, removes ATG, forward
stdapdh-R	AGAAGAGAuTCAAACCAGTTTACGGATCCACG	Amplification of *stdapdh* for USER cloning into pET28a(+)-TEV in-phase with Nt His-tag, reverse
slalr-F	AGGGCCAuAATGAAACACCTACGCGTGT	Amplification of *slalr* for USER cloning into pET28a(+)-TEV in-phase with Nt His-tag, removes ATG, forward
slalr-R	AGAAGAGAuTCAACCACCCAGGTAAACG	Amplification of *slalr* for USER cloning into pET28a(+)-TEV in-phase with Nt His-tag, reverse
ecalr-F	AGGGCCAuCAGGCGGCGACCGTTGTTAT	Amplification of *ecalr* for USER cloning into pET28a(+)-TEV in-phase with Nt His-tag, removes ATG, forward
ecalr-R	AGAAGAGAuTCAGTCAACGTATTTCATCGCAACAC	Amplification of *ecalr* for USER cloning into pET28a(+)-TEV in-phase with Nt His-tag, reverse
psefdh-F	AGGGCCAuGCTAAAGTTCTGTGCGTTCTG	Amplification of *psefdh* (both parental and engineered) for USER cloning into pET28a(+)-TEV in-phase with Nt His-tag, removes ATG, forward
psefdh-R	AGAAGAGAuTTAAACAGCTTTTTTGAATTTAGCAGCTTC	Amplification of *psefdh* (both parental and engineered) for USER cloning into pET28a(+)-TEV in-phase with Nt His-tag, reverse

[a] The uracil residues relevant for USER cloning are indicated in lowercase.

Table S3. Amino-acid sequences, molar extinction coefficient and molecular weight of the enzymes of this work.

Name	Sequence<br>(*N-terminus→C-terminus*)	ε_{280}<br>(M^{-1} cm^{-1})	MW<br>(kDa)
^{Vp}ALD	MGSSHHHHHHSSGENLYFQGHIIGVPKEIKNHEYRVGMIPASV<br>RELISHGHQVFVETNAGAGIGFSDDDYIAVGASILPTAAEVFA<br>QADMIVKVKEPQAVERAMLKEGQILFTYLHLAPDFPQTEDLIK<br>SKAVCIAYETVTDNMGRLPLLAPMSEVAGRMSIQAGAQTLEKS<br>HGGRGLLLGGVPGVEPAKVVIVGGGVVGANAARMAVGMRADVT<br>ILDRNIDTLRKLDEEFQGRAKVVYSTEDAIEKHVLAADLVIGA<br>VLIPGAAAPKLVTKEHIAKMKPGAAVVDVAIDQGGCFETSHAT<br>THADPTYIVDDVVHYCVANMPGAVARTSTFALNNATLPYIVKL<br>ANKGYREALLADHGFLEGLNVIHGKVTCKEVAEAFNLEYVQPE<br>TAIAMFN	16,390	42.077
StDAPDH	MGSSHHHHHHSSGENLYFQGHDKLRVAVVGYGNVGRYALEAVQ<br>AAPDMELVGVVRRKVLAATPPELTGVRVVTDISQLEGVQGALL<br>CVPTRSVPEYAEAMLRRGIHTVDSYDIHGDLADLRRRLDPVAR<br>EHGAAAVISAGWDPGTDSIIRALLEFMAPKGITYTNFGPGMSM<br>GHSVAVKAIPGVRDALSMTIPAGMGVHKRAVYVELEPGADFAE<br>VERAIKTDPYFVRDETRVTQVESVSALMDVGHGVVMERKGVSG<br>ATHNQLFRFEMRINNPALTAQVMVAALRAAARQKPGCYTMIEI<br>PVIDYLPGDREAWIRKLV	25,900	34.717
^{Sl}ALR	MGSSHHHHHHSSGENLYFQGHNETPTRVYAEIDLDAVRANVRA<br>LRARAPRSALMAVVKSNAYGHGAVPCARAAQEAGAAWLGTATP<br>EEALELRAAGIQGRIMCWLWTPGGPWREAIETDIDVSVSGMWA<br>LDEVRAAARAAGRTARIQLKADTGLGRNGCQPADWAELVGAAV<br>AAQAEGTVQVTGVWSHFACADEPGHPSIRLQLDAFRDMLAYAE<br>KEGVDPEVRHIANSPATLTLPETHFDLVRTGLAVYGVSPSPEL<br>GTPAQLGLRPAMTLRASLALVKTVPAGHGVSYGHHYVTESETH<br>LALVPAGYADGIPRNASGRGPVLVAGKIRRAAGRIAMDQFVVD<br>LGEDLAEAGDEAVILGDAERGEPTAEDWAQAAHTIAYEIVTRI<br>GGRVPRVYLGG	58,900	42.164
^{Ec}ALR	MGSSHHHHHHSSGENLYFQGHQAATVVINRRALRHNLQRLREL<br>APASKMVAVVKANAYGHGLLETARTLPDADAFGVARLEEALRL<br>RAGGITKPVLLLEGFFDARDLPTISAQHFHTAVHNEEQLAALE<br>EASLDEPVTVWMKLDTGMHRLGVRPEQAEAFYHRLTQCKNVRQ<br>PVNIVSHFARADEPKCGATEKQLAIFNTFCEGKPGQRSIAASG<br>GILLWPQSHFDWVRPGIILYGVSPLEDRSTGADFGCQPVMSLT<br>SSLIAVREHKAGEPVGYGGTWVSERDTRLGVVAMGYGDGYPRA<br>APSGTPVLVNGREVPIVGRVAMDMICVDLGPQAQDKAGDPVIL<br>WGEGLPVERIAEMTKVSAYELITRLTSRVAMKYVD	40,910	41.427
NAD-^{Pse}FDH	MGSSHHHHHHSSGENLYFQGHAKVLCVLYDDPVDGYPKTYARD<br>DLPKIDHYPGGQTLPTPKAIDFTPGQLLGSVSGELGLRKYLES<br>NGHTLVVTSDKDGPDSVFERELVDADVVISQPFWPAYLTPERI<br>AKAKNLKLALTAGIGSDHVDLQSAIDRNVTVAEVTYCNSISVA<br>EHVVMMILSLVRNYLPSHEWARKGGWNIADCVSHAYDLEAMHV<br>GTV**A**AGRIGLAVLRRLAPFDVHLHYT**D**RHRLPESVEKELNLTW<br>HATREDMYPVCDVVTLN**C**PLHPETEHMINDETLKLFKRGAYIV<br>NTARGKLCDRDAVARALESGRLAGYAGDVWFPQPAPKDHPWRT<br>MPYNGMTPHISGTTLTAQARYAAGTREILECFFEGRPIRDEYL<br>IVQGGALAGTGA**HS**YSKGNATGGSEEAAKFKKAV	59,820	46.410
NADP-^{Pse}FDH	MGSSHHHHHHSSGENLYFQGHAKVLCVLYDDPVDGYPKTYARD<br>DLPKIDHYPGGQTLPTPKAIDFTPGQLLGSVSGELGLRKYLES<br>NGHTLVVTSDKDGPDSVFERELVDADVVISQPFWPAYLTPERI	59,820	46.381

	AKAKNLKLALTAGIGSDHVDLQSAIDRNVTVAEVTYCNSISVA EHVVMMILSLVRNYLPSHEWARKGGWNIADCVSHAYDLEAMHV GTV**G**AGRIGLAVLRRLAPFDVHLHYT**Q**RHRLPESVEKELNLTW HATREDMYPVCDVVTLN**A**PLHPETEHMINDETLKLFKRGAYIV NTARGKLCDRDAVARALESGRLAGYAGDVWFPQPAPKDHPWRT MPYNGMTPHISGTTLTAQARYAAGTREILECFFEGRPIRDEYL IVQGGALAGTGA**KV**YSKGNATGGSEEAAKFKKAV		

The His_{6x}-tag and the TEV-site are indicated underlined. The changes in the sequence between NADP-*Pse*FDH and NADP-*Pse*FDH are highlighted in grey. The molar extinction coefficient and the molecular weight were predicted based on the amino acid sequence using the ProtParam tool (https://web.expasy.org/protparam/). The molar extinction coefficient was calculated assuming all the cysteine residues are reduced. ε_{280}, molar extinction coefficient at an absorbance value of 280 nm. MW, molecular weight.

Enzyme assays

Method S1. Racemase activity assay.

The purified racemases were assayed initially against its natural substrate L-Ala (5 mM) to confirm activity. ^{Sl}AlaR and ^{Ec}AlaR were added at 4.8×10^{-3} μM and 1.1×10^{-2} μM, respectively. The racemase activity was coupled with 1 U mL^{-1} D-amino acid oxidase from porcine kidney (catalogue # A5222, Merck) and 1 U mL^{-1} L-lactic dehydrogenase (catalogue # L2500, Merk). Activity was followed by monitoring the oxidation of 0.5 mM NADH during the conversion of Pyr into L-lactate (**Figs. S7 and S8**). Catalase (2 U mL^{-1}) from bovine liver (catalogue # C1345, Merck) was added to prevent the accumulation of hydrogen peroxide.

Method S2. Formate dehydrogenase activity assay.

The activity of ^{Pse}NAD-FDH and ^{Pse}NADP-FDH was verified against formate. The assay was prepared using 10 mM formate and 50 mM of sodium phosphate (pH = 8.0). For ^{Pse}NAD-FDH, 0.5 mM NAD^+ and 1.8×10^{-2} μM enzyme were used, whereas the assay for ^{Pse}NADP-FDH was prepared with 0.5 mM $NADP^+$ and 2.8×10^{-2} μM biocatalyst. The reduction of the cofactor over time was monitored by the change on absorbance at 340 nm (**Fig. S11**).

Supplementary Figures

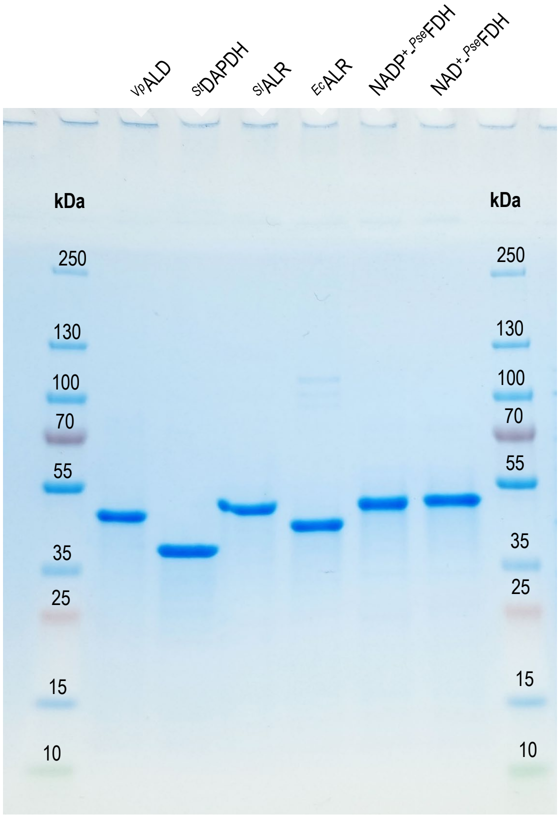

Fig. S1. SDS-PAGE of the enzymes produced and purified in this study. A total amount of 1 μg of protein was loaded per lane.

```
VpALDH      MIIGVPKEIKNHEYRVGMIPASVRELISHGHQVFVETNAGAGIGFSDDDYIAVGASILPT 60
MtALDH      MRVGIPTETKNNEFRVAITPAGVAELTRRGHEVLIQAGAGEGSAITDADFKAAGAQLVGT 60
GkALDH      MIIGVPKEIKNNENRVAITPAGVLSFVQAGHTVLIEKEAGVGSGFNDSDYARAGAQIIER 60
            * :*:*.* **:* **.: **.* .:   ** *:::  ** * .:.* *:  .**.::
VpALDH      AAEVFAQADMIVKVKEPQAVERAMLKEGQILFTYLHLAPDFPQTEDLIKSKAVCIAYETV 120
MtALDH      ADQVWADADLLLKVKEPIAAEYGRLRHGQILFTFLHLAASRACTDALLDSGTTSIAYETV 120
GkALDH      AEDVWAQADMVMKVKEPLPSEYRFFRPGLVLFTYLHLAADPELTRVLKESGVIAIAYETV 120
            * :*:*:**:::*****   *   :: * :***:**** .   *  * .* . .******
```

Fig. S2. Partial alignment of ALD orthologues. ^{Vp}ALD, the ALD from *Mycobacterium tuberculosis* (^{Mt}ALD, PDB ID 2VHW_1) and one ALD from *Geobacillus kaustophilus* (^{Gk3448}ALD, PDB ID 8HYH_1) are aligned showing that the residues involved in the pyruvate binding to active site (Arg15, Lys75 and His96) are conserved.

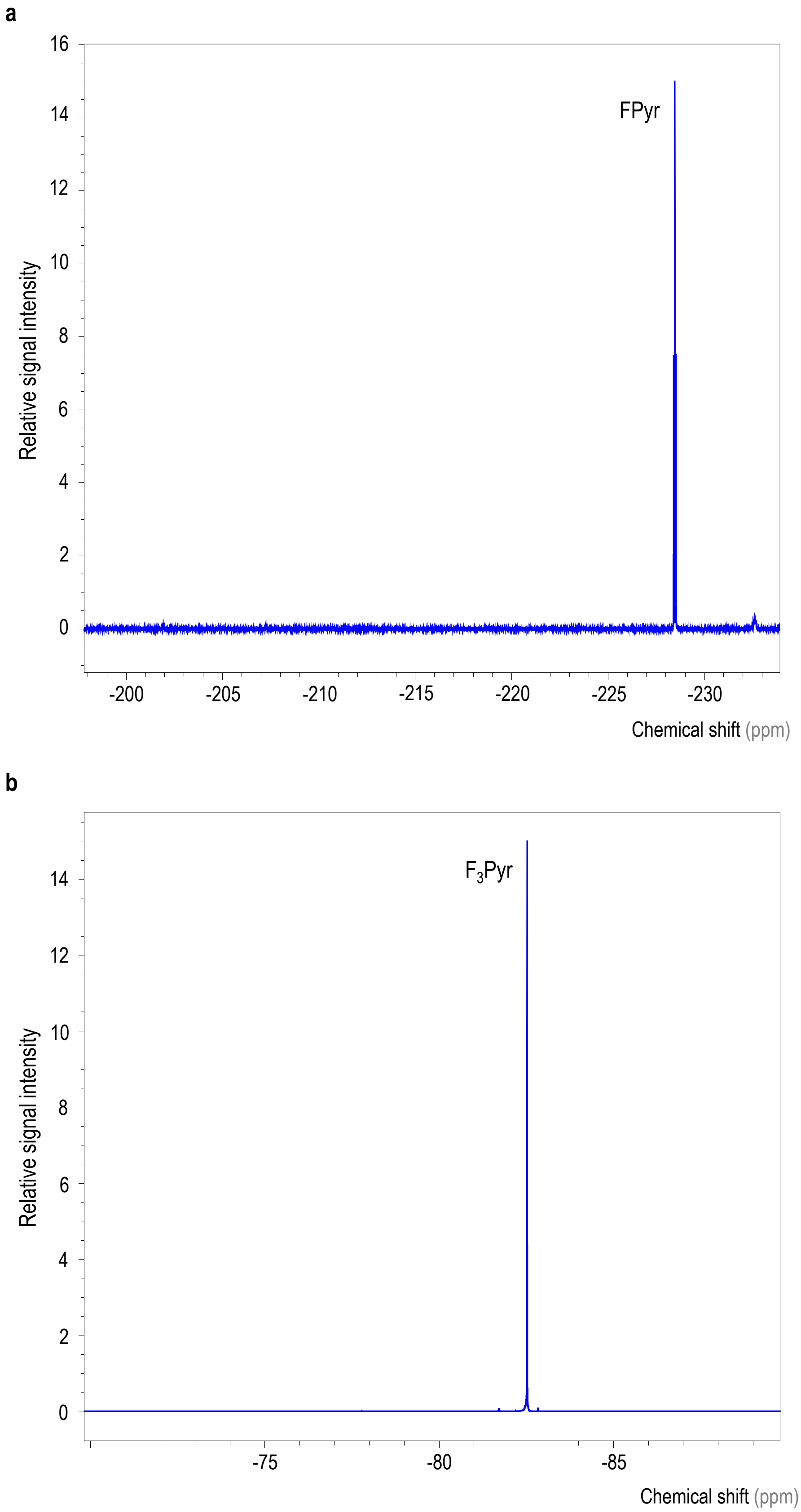

Fig. S3. ^{19}F-NMR spectrum from a reaction mixture prior to the addition of enzyme. a FPyr, prior to the addition of ^{Vp}ALD or StDAPDH to catalyze the corresponding reductive amination (the triplet signal from FPyr is labeled). **b** F_3Pyr prior to the addition of ^{Vp}ALD or StDAPDH to catalyze the corresponding reductive amination (the singlet signal from F_3Pyr is labeled).

a

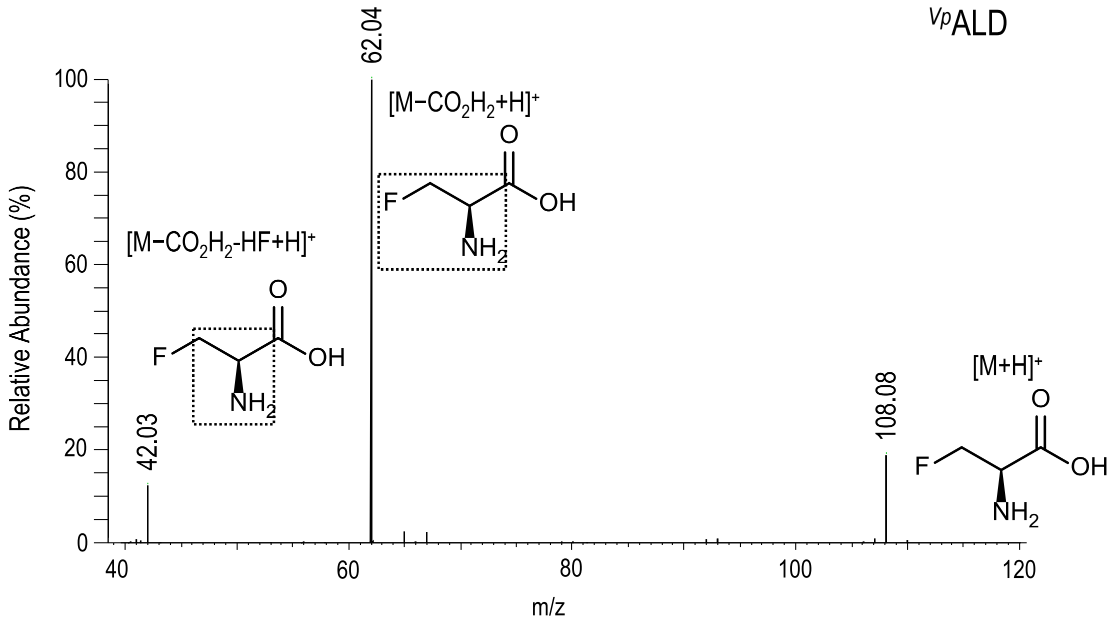

b

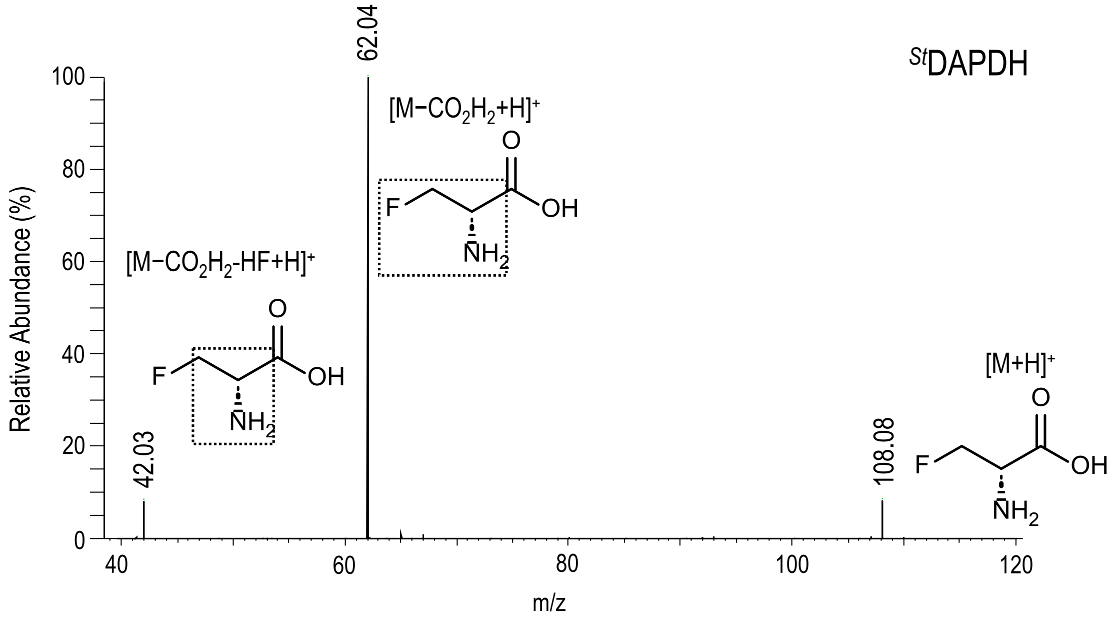

Fig. S4. MS(MS) spectra of FAla enzymatically produced from FPyr. a using ^{Vp}ALD and **b** using StDAPDH. Both spectra show the parent ion (*m/z* = 108.08) and the fragments resulting from losing the carboxylic group (*m/z* = 62.04) and both the carboxylic group and the fluorine substituent (*m/z* = 42.03). The fragment of the parental molecule remaining in the corresponding daughter ion is indicated with a square.

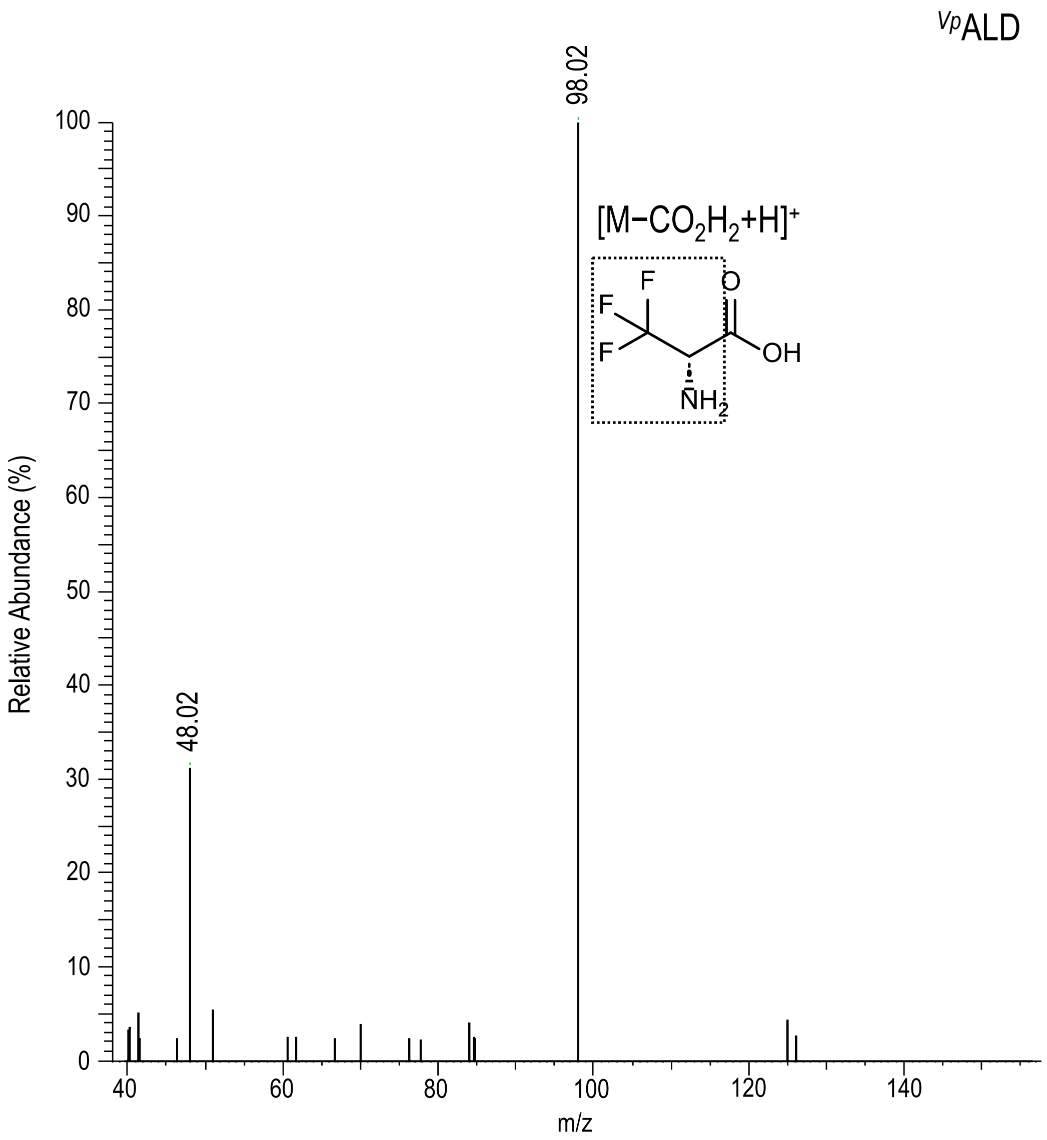

Fig. S5. MS(MS) spectrum of F_3Ala produced by *Vp*ALD from F_3Pyr. The spectrum displays the daughter ion derived from the loss of the carboxylic group. The fragment of the parental molecule remaining in the identified daughter ion is indicated with a square.

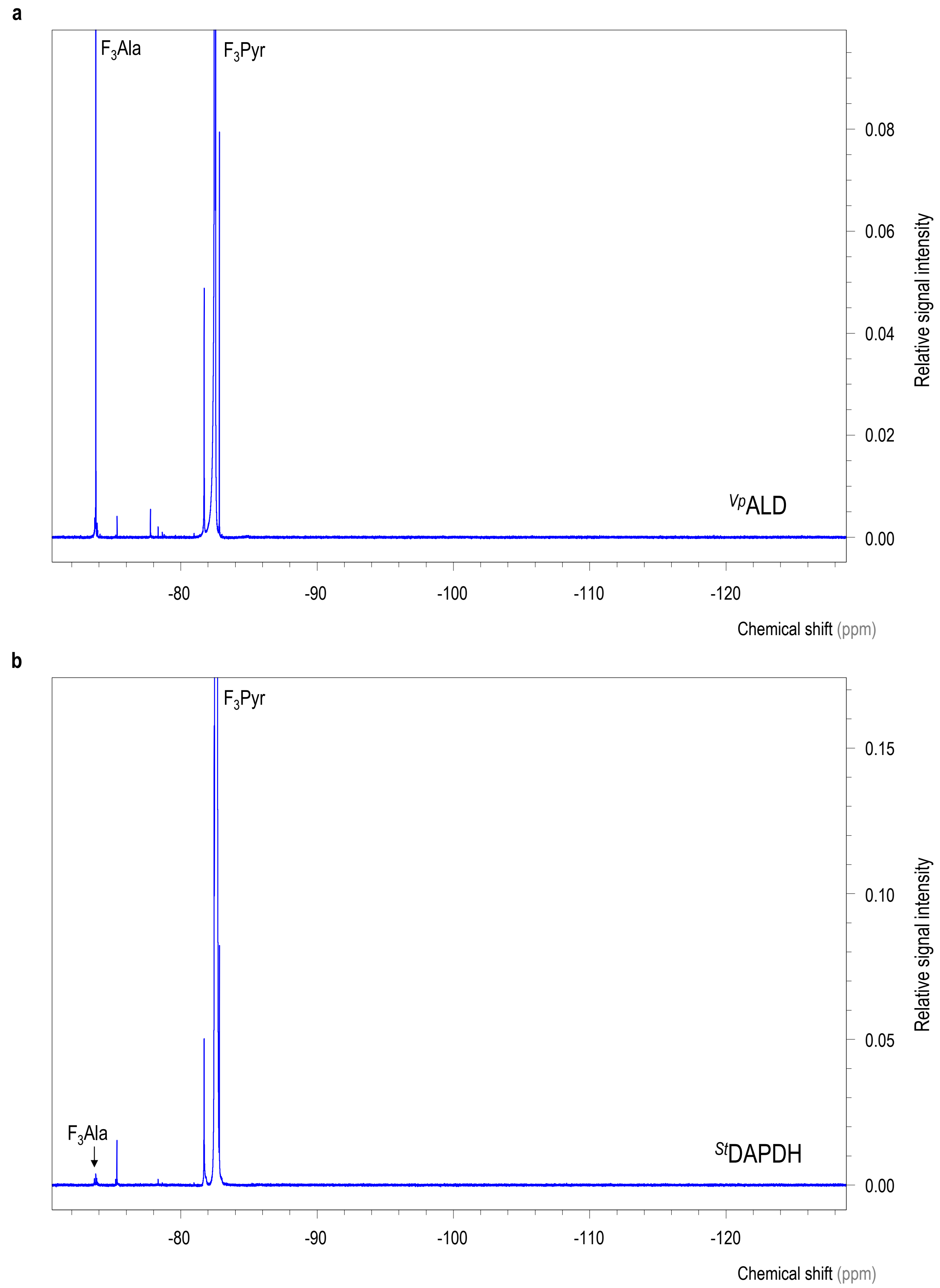

Fig. S6. Representative ^{19}F-NMR spectra for the assays producing F_3Ala. **a** using ^{Vp}ALD and **b** using StDAPDH. The spectra comprise the region from -70 to -130 ppm and are scaled to visualize low intensity signals. No signal of free fluorine was detected at the expected chemical shift of -119 ppm.

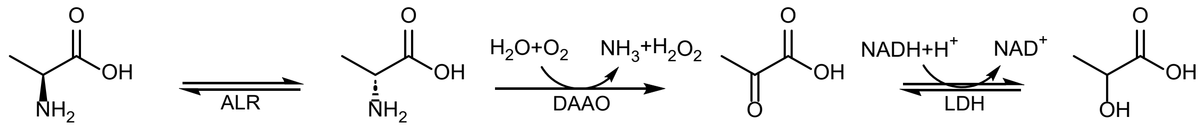

Fig. S7. Racemase activity coupled to lactate synthesis. ALR, alanine racemase; DAAO, D-amino acid oxidase; LDH, L-lactic dehydrogenase.

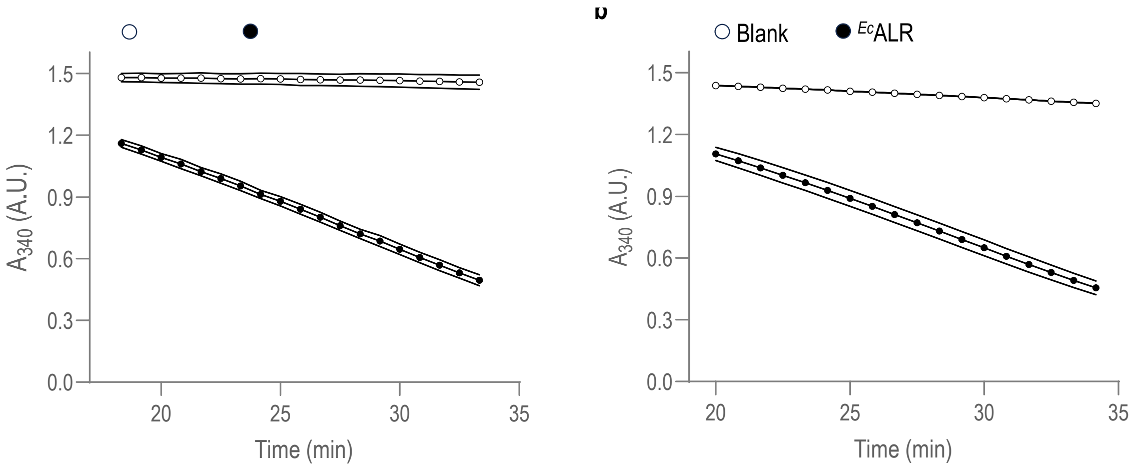

Fig. S8. Activity of the selected ALR against L-Ala over time. a Activity of **^{Sl}ALR** and **b** activity of **^{Ec}ALR** The reaction was monitored by coupling the racemase activity with a DAAO and a lactate dehydrogenase. Read-outs show the oxidation of NADH at A_{340} for both the reaction (*black dots*) and a blank without enzyme (*white dots*). The points represent mean values and the error bars correspond to standard deviations from three independent experiments. *A.U.*, arbitrary units.

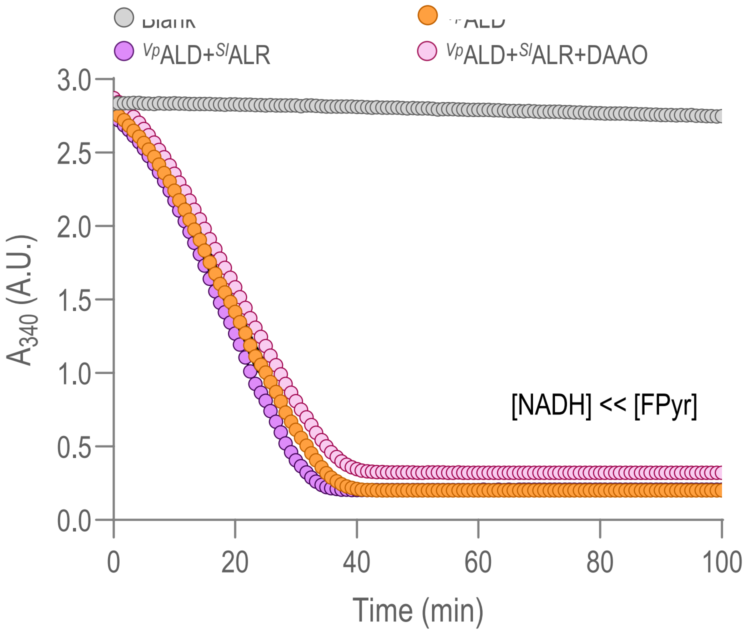

Fig. S9. Spectrophotometric monitoring (A_{340}) of NADH oxidation using FPyr in excess. The reduced cofactor is the limiting reagent and different enzyme combinations are assayed. The assay serves as a control showing that when FPyr is in excess all the tested combinations reach the reaction completion. The points represent mean values from three independent experiments and the error bars correspond to standard deviations from three independent experiments. *A.U.*, arbitrary units.

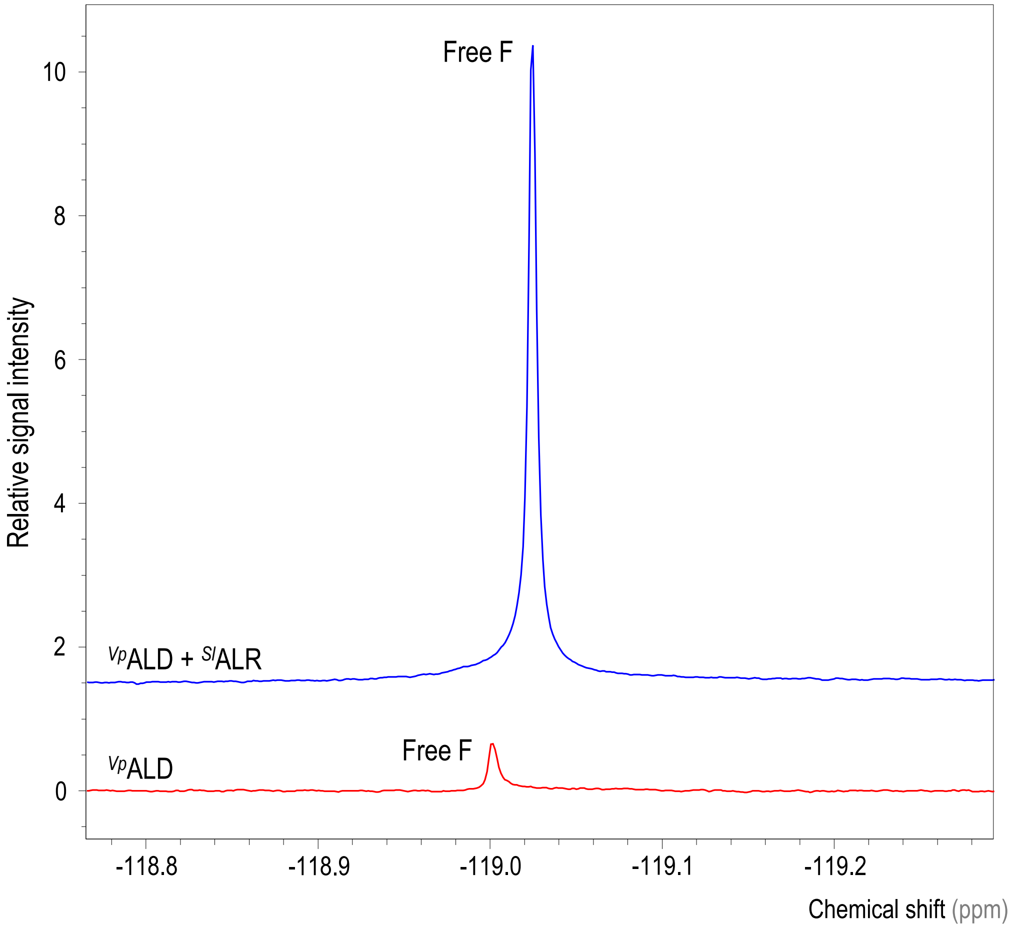

Fig. S10. ^{19}F NMR spectra showing the relative abundance of free F in equivalent reactions catalyzing the reductive amination of FPyr. The red spectrum corresponds to an assay containing ^{Vp}ALD whereas the blue spectrum was obtained from the combination of ^{Vp}ALD and ^{Sl}ALR. Both were recorded after 100 min of reaction and with the same number of scans.

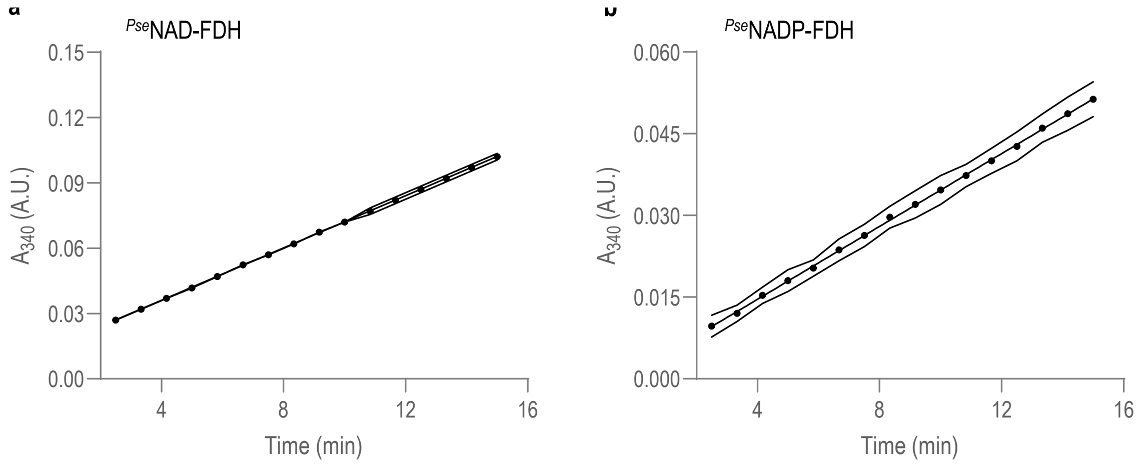

Fig. S11. Activity of the FDH enzymes against formate over time. a ^{Pse}NAD-FDH and **b** ^{Pse}NADP-FDH against formate over time. Read-outs show the reduction of respectively NAD^+ and $NADP^+$ at A_{340}. The points represent mean values and the error bars correspond to standard deviations from three independent experiments. *A.U.*, arbitrary units.

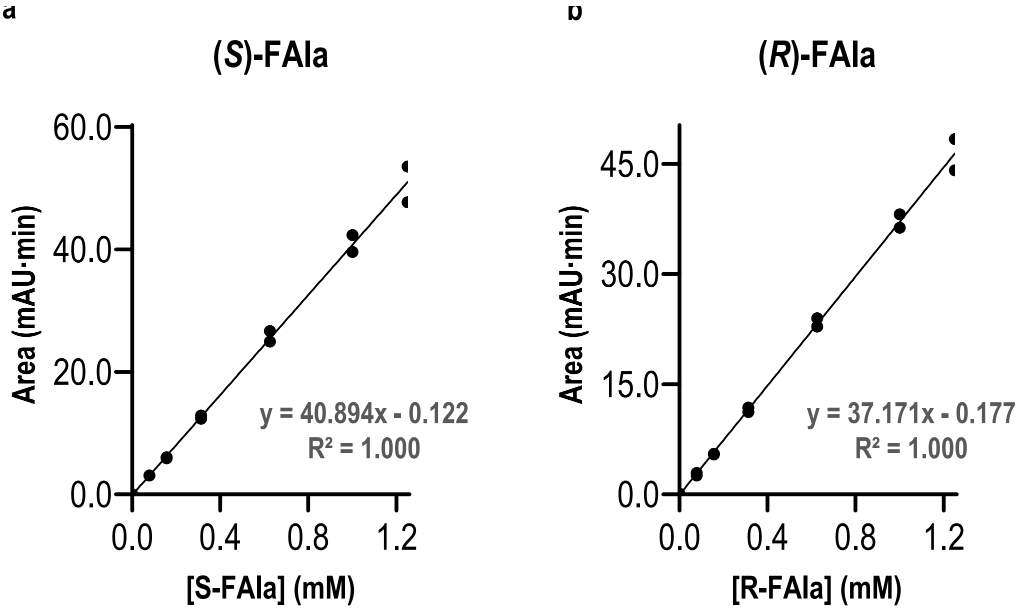

Fig. S12. Calibration curves to determine the concentration of the Ala enantiomers in the reaction samples. a D-Ala and **b** L-Ala. Two series of independent replicates were prepared and analyzed by reversed-phase chromatography. The concentrations of the standards are plotted against the area under the curve. Absorbance was monitored at 310 nm.

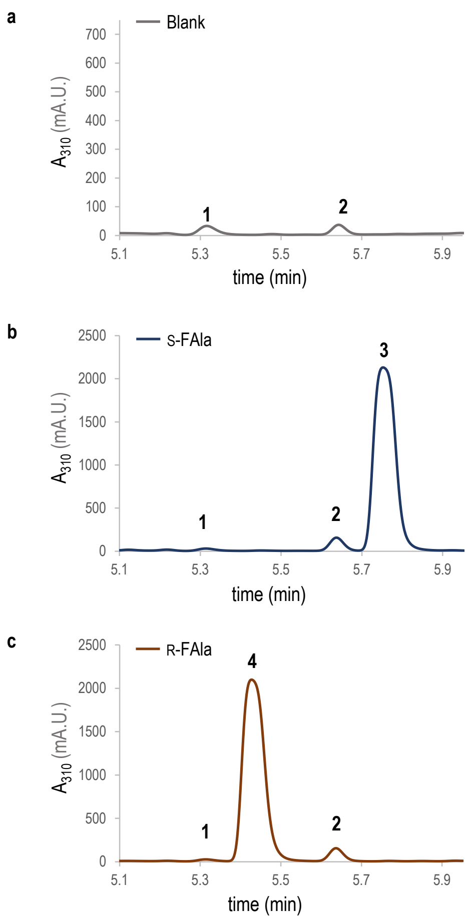

Fig. S13. Representative HPLC profiles of control samples derivatized with the Marfey's reagent. The figure shows the chromatogram corresponding to **a** blank of MiliQ water, **b** standard of (*S*)-FAla and **c** standard of (*R*)-FAla. The peaks of the region of interest are labeled with numbers. 1: Unidentified peak; 2: Unidentified peak; 3: (*S*)-3-fluoroalanine; 3: (*R*)-3-fluoroalanine. m*A.U.*, milli-absorbance units.
