# Supplementary material for: Enzymatic synthesis of mono- and trifluorinated alanine enantiomers expands the scope of fluorine biocatalysis
Source: Commun Chem. 2024 May 9;7:104. doi: 10.1038/s42004-024-01188-1 (PMC11082193; doi:10.1038/s42004-024-01188-1)
Supplement: Supplementary file 3 — Description of Additional Supplementary Files [file 42004_2024_1188_MOESM3_ESM.pdf]

Descriptions of Additional Supplementary Files

Supplementary Data 1

Description: NMR spectra

Supplementary Data 2

Description: Numerical source data for Figures 2a, 2b, 3b, 3c, 3e, 3f, 4d, S8, S9, S11 and S12
