# Supplementary material for: Enzymatic synthesis of mono- and trifluorinated alanine enantiomers expands the scope of fluorine biocatalysis
Source: Commun Chem. 2024 May 9;7:104. doi: 10.1038/s42004-024-01188-1 (PMC11082193; doi:10.1038/s42004-024-01188-1)

Fig. 2d. Representative 19F-NMR (752.83 MHz in H2O/D2O 90/10) spectra for the assays producing FAla using VpALD (orange) and StDAPDH (blue).

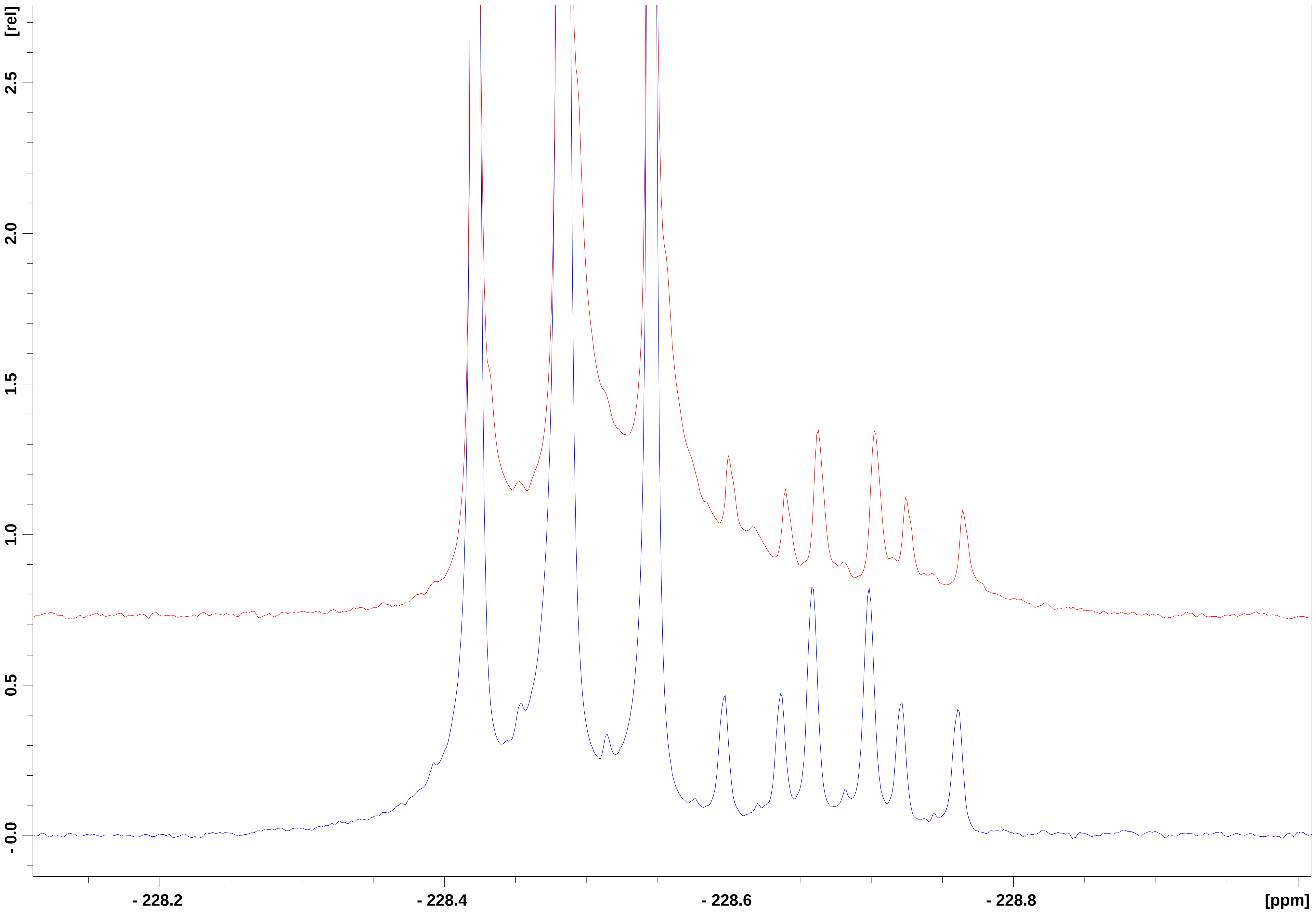

Fig. 2d. Representative 19F-NMR (752.83 MHz in H2O/D2O 90/10) spectra for the assays producing F3Ala using VpALD (orange) and StDAPDH (blue).

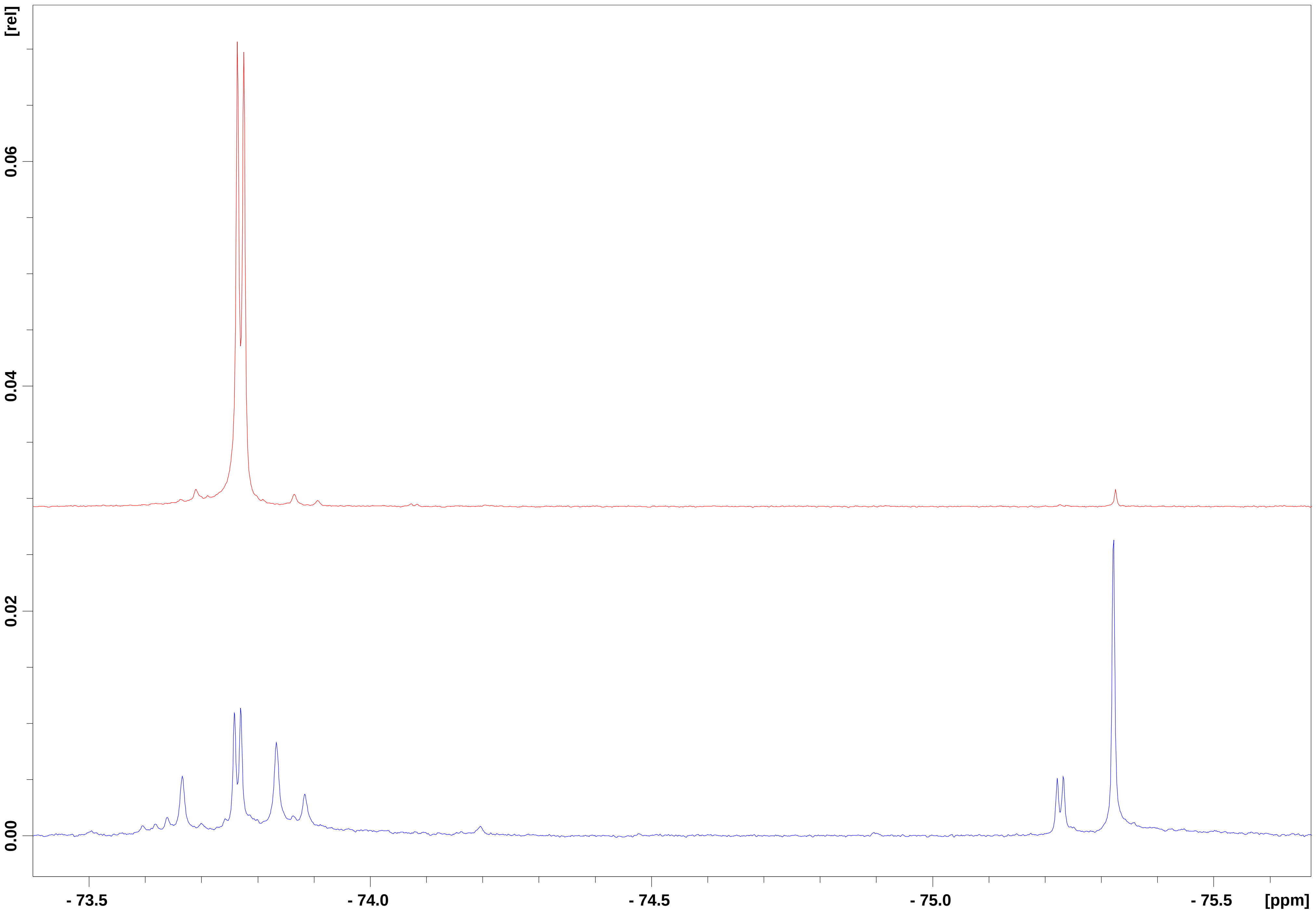

Fig. 6a. Representative 19F-NMR (752.83 MHz in H2O/D2O 90/10) spectra for the assays producing F3Ala using VpALD.

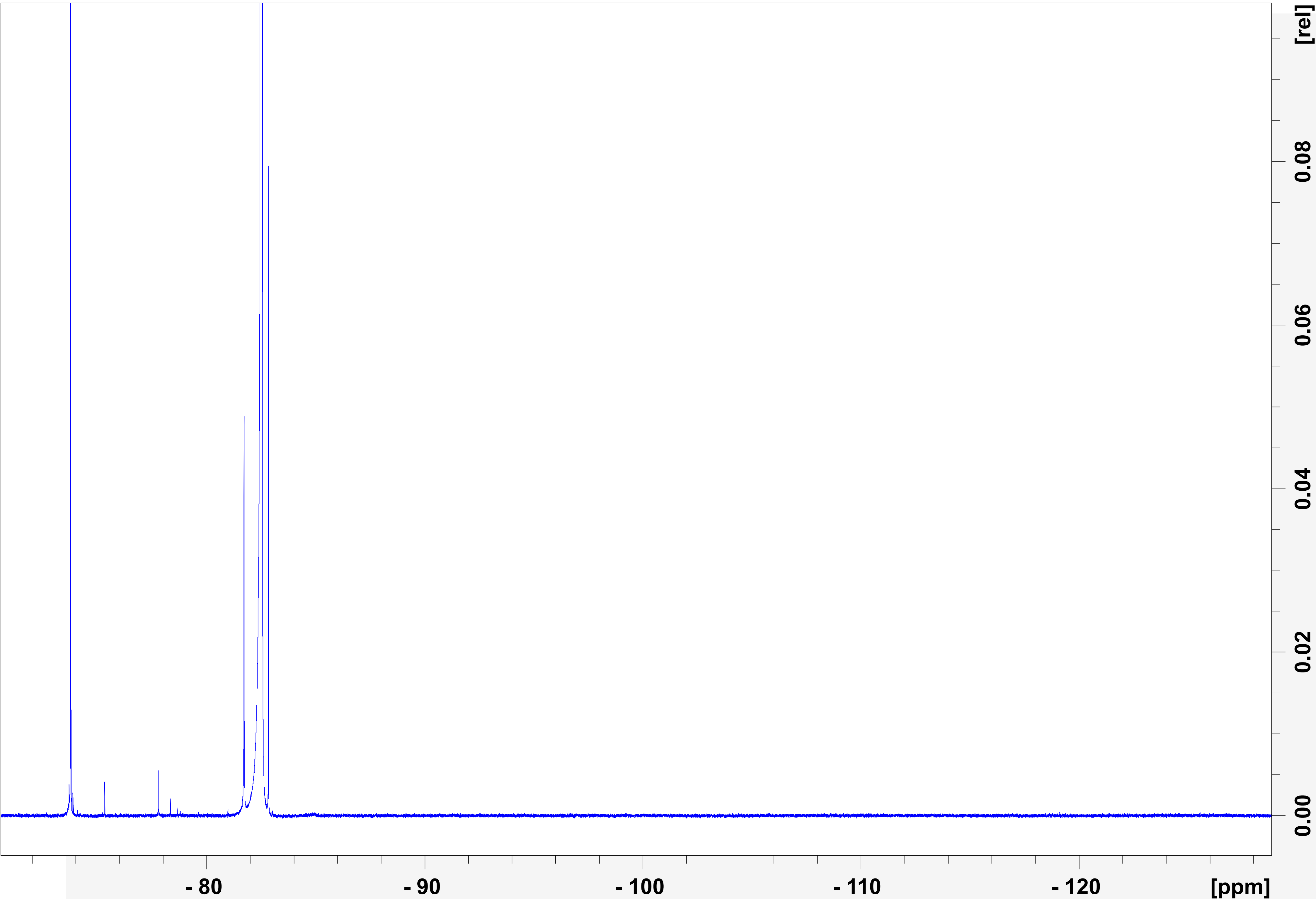

Fig. S6b. Representative 19F-NMR (752.83 MHz in H2O/D2O 90/10) spectra for the assays producing F3Ala using StDAPDH.

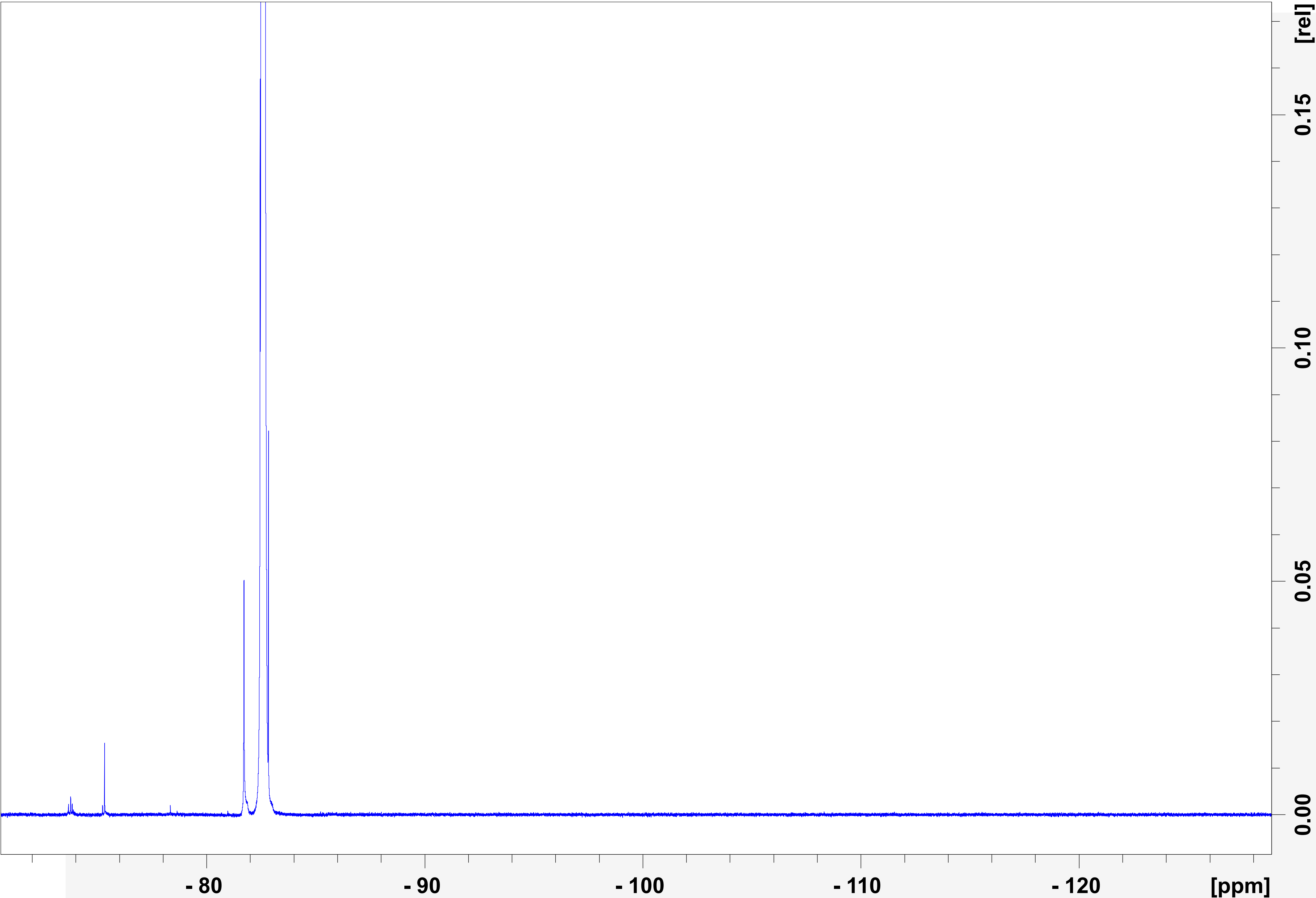

Fig. S10. 19F NMR (752.83 MHz in H2O/D2O 90/10) spectra showing relative abundance of free F in equivalent reactions catalyzing the reductive amination of FPyr using VpALD (red) or the combination of VpALD and SlALR (blue).

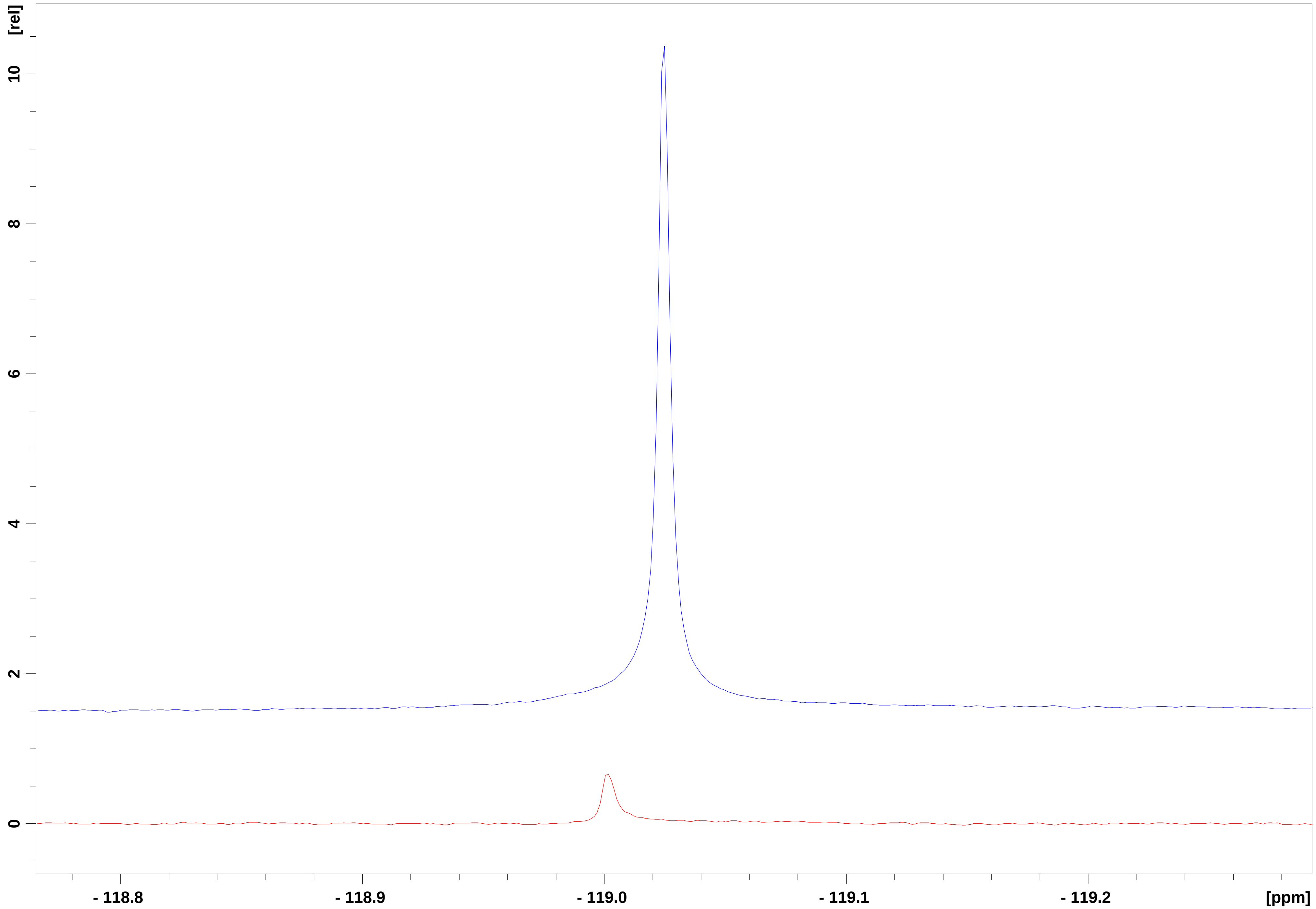

Supplement: Supplementary file 4 — Supplementary Data 1 [file 42004_2024_1188_MOESM4_ESM.pdf]
